# A Convenient Nozzle-Cap for the Urethral Syringe

**Published:** 1883-11

**Authors:** Jno. Thad. Johnson

**Affiliations:** Atlanta, Ga.


### A CONVENIENT NOZZLE-CAP FOR THE URETHRAL SYRINGE.

By JNO. THAD. JOHNSON, M.D., Atlanta, Ga.

A little thing is sometimes worth the mention, because of the frequency with which the littleness may annoy.

When a young man finds it necessary to thrust the long, slender pipe of a syringe into a bottle for the purpose of filling the syringe from the bottle, it is annoying to find the long pipe too short to reach the fiuid, the syringe too large to go into the bottle, and yet not large enough to prevent the flnid from running out if we invert the implements. Nor can such unfortunate often find it convenient to carry with him a wide-mouthed bottle for use as temporary reservoir from which to charge the syringe. He likes to reduce his implements of warfare so as to go in light marching order, especially when "on the wing."

Some time since, while instructing a mercantile traveler as to the method of combating a sequela of a momentary and impulsive indiscretion, this difficulty of fixing the syringe was suggested. An ordinary rubber nursing-bottle nipple was on the table before me; seizing it, I chipped off its tapering extremity, slipped it over the syringe-pipe, demonstrated to the patient how thoroughly the instrument, thus re inforced, drew the fluid from the inverted bottle, and then how softly it could be made to fill the urethral meatus.

This is an almost sacrelegious perversion of the original object for which the nipple was made; a vessel made to honor and cashiered to dishonor. And, after all, the matter probably scarcely worth the mention; and, if not, Mr. Editor, the yawning waste-basket sits conveniently near you.

